# What is the evidence to support early supervised exercise therapy after primary total knee replacement? A systematic review and meta-analysis

**DOI:** 10.1186/s12891-019-2415-5

**Published:** 2019-01-29

**Authors:** Larissa Nicole Sattler, Wayne Anthony Hing, Christopher John Vertullo

**Affiliations:** 1 Bond University, 14 University Drive, Robina, QLD 4226 Australia; 2 PO Box 8711, Gold Coast, MC, QLD 9726 Australia; 3 Pindara Private Hospital, Allchurch Avenue, Benowa, QLD 4217 Australia; 4 Knee Research Australia, 8-10 Carrara Street, Benowa, QLD 4217 Australia

**Keywords:** Total knee replacement, Physiotherapy, Rehabilitation, Physical therapy specialty, Physical therapy modalities, Exercise therapy, Systematic review and meta-analysis

## Abstract

**Background:**

Total knee replacement (TKR) patients participate in early supervised exercise therapy programs, despite a lack of evidence for such programs or the optimal type, duration or frequency to provide the best clinical outcomes. As hospital stay rates decrease worldwide, the first days after joint replacement surgery are of increasing clinical importance. The purpose of this study was to investigate any reported effects of published early exercise therapy following TKR surgery.

**Methods:**

Databases PubMed, CINAHL, Embase, Cochrane, and Pedro were searched up to August 2018 for trials which investigated an early supervised exercise therapy, commencing within 48 h of surgery. Risk of bias was evaluated using a Modified Downs and Black Checklist and meta-analysis of results was conducted using Review Manager (RevMan). Standardised Mean Differences (SMD) or Mean Differences (MD) and 95% confidence intervals were calculated and combined in meta-analyses.

**Results:**

Four studies (323 patients) that used four different interventions were identified, including Modified Quadriceps Setting, Flexion Splinting, Passive Flexion Ranging and a Drop and Dangle Flexion regime. Patients receiving the Drop and Dangle flexion protocol had superior flexion in the first 2 days after TKR and at discharge, the Flexion Splint patients were discharged earlier and had greater flexion at 6-weeks postoperatively, and the Modified Quadriceps Setting group showed greater hamstring and gluteal muscle strength. Results of the methodological quality assessment showed included studies were of moderate quality. The meta-analysis included 3 of the 4 trials and found no significant differences between groups in maximum knee flexion (MD = 1.34; 95% CI, − 5.55–8.24) or knee society scores (MD = − 1.17; 95% CI, − 4.32–1.98) assessed at 6 weeks post-operatively.

**Conclusion:**

The paucity and heterogeneity of existing studies that examine early supervised exercise therapy following TKR surgery makes it challenging for clinicians to deliver high-quality evidence-based exercise programs in the early postoperative period. Although superior knee flexion range was found across differing regimes, the meta-analysis showed no significant difference in this outcome between groups at 6 weeks. The results of this review show high quality randomized clinical trials are urgently needed to evaluate the impact of early exercise following TKR surgery.

**Trial Registration:**

This review was registered with PROSPERO (CRD42017081016).

**Electronic supplementary material:**

The online version of this article (10.1186/s12891-019-2415-5) contains supplementary material, which is available to authorized users.

## Background

Worldwide rates of primary total knee replacement (TKR) as a treatment for end stage knee osteoarthritis are increasing between 5 and 17% per year [1]. By 2025, knee osteoarthritis (OA) prevalence is expected to increase by 40% and the most common surgical intervention for end-stage knee OA is TKR [2]. The total hospital cost for knee replacement for osteoarthritis nearly tripled during 2002–2013 in the United States, amounting to $12.0 billion, this trend is expected to continue in accordance with the aging populations and rising obesity rates [3].

In contrast to this rapid rise in the number of TKR procedures, hospital length of stay (LOS) rates after TKR surgery are declining. In the United States, from 2002 to 2013, the mean inpatient period post TKR decreased from 4.06 to 2.97 days and the percentage of hospital inpatient periods of ≥5 days decreased from 24.7 to 6.1% in 2013 [3]. Reduction in LOS following TKR can reduce the economic burden of knee osteoarthritis, and evidence now demonstrates that factors such as the use of clinical pathways, advances in blood management, multimodal analgesia and early ambulation can all contribute to this reduction [4–7].

Due to this decrease in length of stay, and emphasis on as early ambulation after surgery as possible, it is important to examine early post-operative inpatient exercise interventions [8–12]. These interventions can be separated into passive interventions, such as cold therapy, compression or continuous passive motion, and, the target of this review, supervised exercise therapy conducted by a physiotherapist in an acute in-patient setting.

One purpose of early postoperative physiotherapy following TKR is to prepare patients for discharge following their operation. As a result of shorter length of stays, inpatient physiotherapy has become increasingly concentrated on early and safe mobility, with accelerated rehabilitation pathways becoming the standard of care [13].

This inpatient therapist directed physiotherapy usually involves active exercises to improve knee range of motion and muscle strengthening [14, 15]. However, despite the majority of TKR patients receiving an in-hospital physiotherapy program of some type, there are limited studies to demonstrate either its effectiveness or the optimum program design [16]. The recent meta-analysis by Artz et al. examined the effectiveness of post-discharge physiotherapy exercise in patients with primary total knee replacement, in comparison to our study, which focused on programs implemented in an acute inpatient setting [14]. Large variations between institutions and individual clinicians exist as to what type of active inpatient therapy is prescribed, and its duration and its frequency, with only gait retraining and exercise prescription being frequently utilized [15]. This variation can result in suboptimal outcomes at a greater cost.

The purpose of this study was to investigate any reported effects of published early exercise therapy following total knee replacement surgery.

## Methods

This systematic review and meta-analysis was prospectively registered on PROSPERO (International prospective register of systematic reviews), registration CRD42017081016. The review was reported in accordance with the guidelines from the PRISMA (Preferred Reporting Items for Systematic Reviews and Meta-Analyses) statement [17].

### Search strategy

Relevant published studies were extracted for analysis by the primary investigator from PubMed, Cumulative Index to Nursing and Allied Health (CINAHL), Embase, Cochrane, and Pedro. Key terms were identified for the search, including knee replacement, physiotherapy, and rehabilitation, as well as synonym words. The search strategy included applying wildcards (*), also known as truncation symbols, which represent one or more characters when able, using Boolean Operators ‘and’ or ‘or’ to combine the search key search terms, and searching up to August 2018 to collect the best current evidence. The complete search strategy is presented in Table 1.

**Table 1:** Critical review databases and search terms

Database	Search Terms		
PubMed CINAHL Embase COCHRANE PEDro	“Arthroplasty, Replacement, Knee” (MESH) OR Knee Replacement OR TKR	AND	“Physical therapy Modalities” (MESH) OR Physical therapy OR “Rehabilitation” (MESH) OR Rehabilitation

### Eligibility criteria

We included studies investigating supervised exercise therapy following TKR in the acute inpatient hospital period. Interventions including electrical stimulation, acupuncture, cryotherapy or electrical modalities such as continuous passive motion (CPM) were excluded as these were considered as an adjunct to physiotherapist led exercise-based interventions. Journal articles were the primary source collected and search results were filtered to include randomized controlled and quasi-experimental trials. Manual searches of reference lists within journal articles meeting the inclusion criteria were conducted to ensure all relevant studies were included. Were included and reviewed.

#### Inclusion criteria

- Articles available in full text
- Articles in English
- A key term was required in the study Title or Abstract
- Study design was a randomized controlled or quasi-experimental trial
- A Therapist-led exercise intervention. CPM therapy was not included in this study as the experimental intervention, however, could be part of a patient’s standard care.
- Study setting: exercise intervention commenced in the acute hospital period within 48 h of TKR surgery and prior to discharge from the inpatient setting
- Participants were post-operative primary unilateral total knee replacement patients

### Study selection

Based on the inclusion criteria, an initial screening of titles and abstracts occurred to isolate possible relevant papers. Next, a screening of extracted full text papers was conducted for final review. The primary investigator, screened all titles, abstracts, full papers and made the decision about study eligibility. Those studies that were included in this literature review were then screened for eligibility to be included for meta-analysis based on similarity of reported outcomes.

### Data extraction

A data extraction form was based on the Cochrane Consumers and Communication Review Group data extraction template [18]. One reviewer extracted the data, and it included information on authors, year of publication, location, number of participants and participant features, study setting, interventions and controls used, primary and secondary outcome measures, follow up intervals, adherence and loss to follow up, result findings and adverse events that occurred.

### Methodological quality

The Modified Methodological Quality Checklist by Downs and Black (1998) was used for both randomized controlled trials and non-randomized controlled trials to assess the risk of bias of the included studies [19]. The methodological quality assessment was completed by two independent reviewers, any disagreements were resolved by discussion and consensus or by consultation with a third reviewer if necessary.

The modified Downs & Black checklist used was a twenty-seven-point scale consisting of five subscales (reporting, external validity, internal validity bias, internal validity confounding, and power) to analyse both randomized and nonrandomized controlled trials (Additional file 1). The Downs and Black scale has high internal consistency (r = .89) and criterion validity (r = .90), good test-retest reliability (r = .88) and inter-rater reliability (r = .75) [20]. The studies were rated as poor if they scored 7 or less, limited if they scored 7–13, moderate if they scored 14 to 20 and strong if they scored 21 or greater [21–23]. The quality of the studies was considered in the analysis of the results.

### Statistical analysis

When an outcome was reported in at least two studies, analysis of quantitative data for meta-analysis was completed using a computer software program developed by The Cochrane Collaboration, Review Manager (RevMan, version 5.3) [24]. Effect sizes for eligible outcomes Maximum Knee Flexion, Knee Society Score, and Knee Society Function Score were calculated using mean differences (MD), each with 95% confidence intervals (CIs). A random effects model was used in our analysis to allow for differences in the treatment effects between trials.

Heterogeneity of included studies’ estimates were assessed by computing the I^2^ values and was considered statistically significant at *P* < 0.10. I^2^ values were used to describe the percentage of total variation across studies, an I^2^ value of 25% was considered low, 50% moderate, and 75% high [25]. Variance between outcome measures was estimated using the standard deviation (SD) of the MD between two assessment time-points. If the SD was not reported, we used the SD calculated from the *P*-value for the differences between mean values in the groups.

## Results

### Search, screening, and selection results

**Fig. 1:**
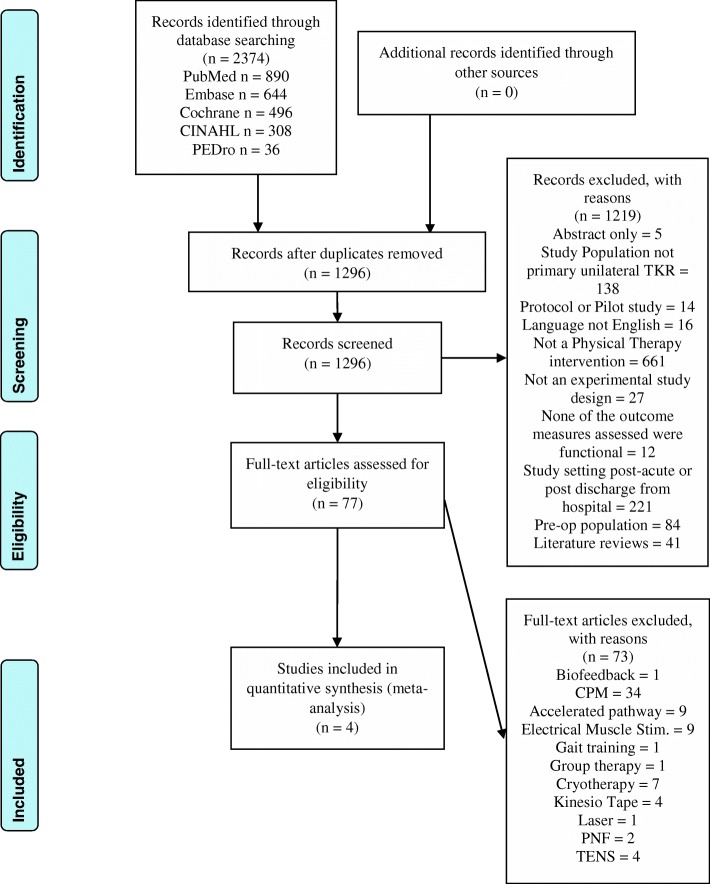
Prisma Flow Diagram of systematic search, screening and selection process

The results of the search strategy and screening process are shown as a flowchart in Fig. 1. Initially, a total of 2374 records were identified from database searching, a manual search of references of the studies that were included did not elicit any further eligible studies. After duplicates were removed, the remaining 1296 articles were screened, after 1219 articles were considered ineligible, 77 articles were assessed in full text. From these articles, a final 4 articles were considered eligible and included in this review. Of those 4 articles, 3 met the inclusion criteria to undergo meta-analysis.

### Description of included studies

The 4 included studies that were reviewed based on the eligibility criteria varied in design, two studies were randomized controlled trials [26, 27] and two studies were of quasi-experimental design [28, 29].

### General characteristics of participants

There were 323 participants who contributed to the studies reported in this review, however, in one study which included 50 participants [27] they used the contralateral limb for alternate group allocation, so there were in total 373 knees of participants included in the final analysis. Total number of participants in the control groups were 179 and in the intervention groups were 194, the gender of the included subjects was predominantly female, 79 and 78% in the control and interventional groups respectively. Mean age was similar between all studies and the same when averaged across the control and intervention groups at 69 years. Table 2 provides a summary of participant characteristics across each study including age, gender, and inclusion and exclusion criteria and Table 3 summarizes each study’s numbers including reported losses to follow up.

**Table 2:** Characteristics of participants

Author, Year, Study Location	Title	No. of Participants		Gender (M/F)		Mean Age & Range if reported (Years)		Inclusion Criteria	Exclusion Criteria
Dujin, P., Jeonghee, K., & Hyunok, L. (2012). Busan, Korea	Effectiveness of Modified Quadriceps Femoris Muscle Setting Exercise for the Elderly in Early Rehabilitation after Total Knee Arthroplasty	Cont	Exp	Cont	Exp	Cont	Exp	TKR patients, Walk alone with/without cane, FFD < 10 deg., Able to communicate, No disease in parts of body except knee joint	Not provided
		22	22	M 0 F 22	M 0 F 22	70.3	69.1		
Hewitt, B., & Shakespeare, D. (2001). Warwickshire, United Kingdom	Flexion vs. extension: a comparison of post-operative total knee arthroplasty mobilization regimes	Cont	Exp	Cont	Exp	Cont	Exp	Unilateral TKR patients	No exclusion criteria
		74	86	M 33 F 41	M 41 F 45	71.7	73.4		
Kim, T., Park, K., Yoon, S., Kim, S., Chang, C., & Seong, S. (2009). Seoul, Korea	Clinical value of regular passive ROM exercise by a physical therapist after total knee arthroplasty	Cont	Exp	Cont	Exp	Cont	Exp	Primary diagnosis of OA, undergoing staged bilateral TKRs, no prior surgery to the knees, normally functioning hip joints,	Unilateral TKR, Wound Complication
		50	*50	M 0 F 50	M 0 F 50	67.9 (53–83)	67.9 (53–83)		
Pongkunakorn, A., & Sawatphap, D. (2014). Lampang, Thailand	Use of drop and dangle rehabilitation protocol to increase knee flexion following total knee arthroplasty: a comparison with continuous passive motion machine	Cont	Exp	Cont	Exp	Cont	Exp	Unilateral TKR patients	FFD > 30 deg., Uncooperative patients, Intraoperative complication preventing early knee motion
		33	36	M 4 F 29	M 2 F 34	68.5 (54–82)	67.0 (57–81)		
Totals		179	194	M 37 F 142	M 43 F 151	69.6	69.3		

*Used contra-lateral limb

**Table 3:** Study numbers

Author	Eligible for Inclusion	Excluded	Number Allocated		Excluded post allocation	Lost to follow up	Included in final analysis	
			Cont	Exp			Cont	Exp
Dujin, P., Jeonghee, K., & Hyunok, L.	44	0	22	22	0	0	22	22
Hewitt, B., & Shakespeare, D.	160	0	74	86	0	0	74	86
Kim, T., Park, K., Yoon, S., Kim, S., Chang, C., & Seong, S.	106	6	50	50	0	0	50	50
Pongkunakorn, A., & Sawatphap, D	86	0	41	45	10	7	33	36
Totals	396	6	187	203	10	7	179	194

### Exercise therapy interventions

All interventions examined in the studies were some form of therapist directed exercise therapy that began within 48 h following TKR surgery. The four interventions included in this review were Modified Quadriceps Setting [26], Flexion Splinting [28], Passive Flexion Ranging [27] and a Drop and Dangle Flexion regime [29], individually these interventions and their respective control groups are described in detail in Table 4.

**Table 4:** Intervention comparison of studies reviewed

Author	Control	Experimental	Delivery of Intervention		
			Timing	Frequency	Duration
Dujin, P., Jeonghee, K., & Hyunok, L.	Conventional Quadriceps Setting (CQS) Protocol Supine position, operated limb in knee extension & ankle dorsiflexion, with a 10s isometric quadriceps contraction CPM daily for 1 h until week 2 post-op. Week’s 2–4 add resistance training for knee flexor and extensor muscles and cycling 1 h/day, 5 times a week.	Modified Quadriceps Setting (MQS) Protocol Seated position (90-degree hip & knee angle) operated limb performed 10s isometric quadriceps contraction with 2 kg sandbag on the other ankle. CPM daily for 1 h until week 2 post-op. Week’s 2–4 add resistance training for knee flexor and extensor muscles and cycling 1 h/day, 5 times a week.	Cont Daily Exp Daily	Cont 10 Repetitions Exp 10 Repetitions	Cont 3 sets with 1 min breaks Exp 3 sets with 1 min breaks
Hewitt, B., & Shakespeare, D.	Extension Splint Protocol Immediately post-op, knee placed out straight in a knee immobiliser splint remaining on overnight. Multi-exercise regime with physio commenced day 1 including knee flexion exercises. Knee extension splint only night of day 1, then no longer applied. Exercises continued daily until discharge.	Flexion Splint Protocol Immediately post-op, knee placed on a 90-degree splint remaining on overnight. Physio day 1, 2hrly knee flexed 90d and placed on the 90d splint for 10 mins, the knee was then allowed to straighten out and hang in passive extension for 10 min with ankle supported on a foam block. Multi-ex regime also added to physio day 1. From day 2 the flexion block regime was ceased once the knee could be actively flexed to 90 degrees, the same standard was set for the night flexion splint.	Cont Daily Exp Daily	Cont Once Exp 2-hrly until active KROM = 90d after day 2	Cont Overnight day 0–1 Exp 2-hourly and overnight
Kim, T., Park, K., Yoon, S., Kim, S., Chang, C., & Seong, S.	No-PROME Protocol Day 0 = quad’s strength ex Day 1 = 50 mins CPM 0–30 degrees + gait training, CPM ROM gradually increased over next two weeks Day 2 = Same as 1 & 2 and add Drop/Dangle + active knee ROM ex’s Day 3–14 = Physio once daily in the rehab centre	PROME Protocol 40 min of physiotherapy: First 20 = quad’s strength + gait training Second 20 = PROME ex (pt placed in supine, 5 mins thigh/calf massage, then PROME routine consisted of holding leg in ext. for first 5 s, then max tolerated flexion for 5 s, one cycle of this took ~20s and 40–50 cycles were performed.	Cont Daily Exp Daily	Cont Once Exp Once	Cont 50 mins Exp 40 mins
Pongkunakorn, A., & Sawatphap, D	CPM Protocol Bandaged in extension post-op and removed day 1 and placed on CPM 0–60 degrees. ROM was increased by 15 degrees or more each day unless not tolerated, progressively increased to115d. Both groups received the same other ROM ex’s and quad strengthening program.	Drop and Dangle Protocol Placed in 70-degree flexion splint post-op then removed day 1. D&D day 1 in a seated position, maximal passive overpressure with the other foot then held for 10s. Then actively assisted into extension by other foot. Both groups received the same other ROM ex’s and quad strengthening program.	Cont Daily Exp Daily	Cont 3 times Exp 3 times	Cont 1 h Exp 1 h

*Cont* Control, *Exp* Experimental

### Outcomes

The primary and secondary outcome measures were varied amongst the included studies. Across the 4 studies, validity and reliability of the selected outcome measures were high. In terms of timing and frequency of the included outcome measures, 3 of the 4 studies took a pre-operative measure to determine baseline [26–28]. The maximum follow-up time for an outcome measure varied significantly between studies with the longest reported follow up of an outcome measure being 1 year [29]. Details of each outcome measure included in the studies reviewed are detailed in Table 5.

**Table 5:** Outcome measures for studies reviewed

Author	Outcome Measure(s)	How Outcome was measured	Validity/Reliability	Frequency of Outcome	Adverse Events
Dujin, P., Jeonghee, K., & Hyunok, L.	1) Muscle strength: Quadriceps, Hamstrings, Gluteus Maximus [31] 2) 6 Minute Walk Test [32]	1) Handheld dynamometer 2) Distance (m)	All outcomes are valid & reliable	All outcomes were measured pre-operatively and at 2 weeks and 4 weeks post-surgery.	Nil reported
Hewitt, B., & Shakespeare, D.	1) Knee Society knee & function scores [33] 2) FFD [34] 3) Max Flex [34] 4) ROM [34] 5) Analgesic Requirements 6) Blood Loss	1) Survey 2) Goniometer 3) Goniometer 4) Goniometer 5) Medical Chart 6) Medical Chart	Outcomes 1–4 are valid and reliable; outcomes 5 & 6 have not been reported by the authors Only Outcome’s 2, 3 & 4 have values reported by the authors, there are no values reported for outcomes 1,5 & 6.	Outcomes 1–4 were measured pre-operatively (1 day prior to OT) and 6 weeks post-surgery; Outcomes 5–6 were measured during admission.	Nil reported
Kim, T., Park, K., Yoon, S., Kim, S., Chang, C., & Seong, S.	1) Knee Society knee & function scores [33] 2) WOMAC scores 3) Flexion contracture [34] 4) Max Flex [34] 5) Patient reported preference of protocol	1) Survey 2) Survey 3) Goniometer 4) Goniometer 5) Survey	Outcomes 1–4 are valid & reliable; Outcome 5 has not been tested for validity or reliability	Outcomes 1 & 2 were measured pre-operatively and 6 months post-surgery; Outcomes 3 & 4 were measured pre-operatively & at 7 days, 14 days, 6 weeks, 3 months and 6 months; Outcome 5 was assessed at day of discharge.	Nil reported
Pongkunakorn, A., & Sawatphap, D	1) OT time 2) Blood Loss 3) LOS 4) Knee Society knee & function scores [33] 5) Passive Flexion ROM [34]	1) Medical Chart 2) Medical Chart 3) Medical Chart 4) Survey 5) Goniometer	All outcomes are valid & reliable	Outcomes 1–4 were measured during admission; Outcome 5 was measured during admission (once daily for 7 days until d/c), and at 6 weeks and 1-year post-surgery.	Nil reported

### Patient reported outcome measures

A survey style patient reported outcome measure (PROM) was included in 3 of the 4 studies [27–29], however, differing PROM tools were used, and results were only reported in 2 of these [27, 29].

### Knee flexion and functional mobility

Maximum knee flexion ROM was also assessed in 3 of the 4 included studies [27–29]. Only 1 of the studies included a functional mobility outcome measure, in this case the 6-min walk test [26].

### Meta-analysis results

**Fig. 2:**
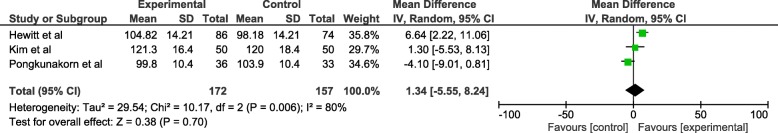
Forest plot diagram of Maximum Knee Flexion at 6 weeks, Exercise Intervention (EI) vs Standard Therapy (ST)

Maximum knee flexion was assessed in 3 of the 4 studies [27–29], 270 knees were measured at 6 weeks post TKR, the results of the meta-analysis are presented in Fig. 2. There was no significant difference between the Exercise Intervention (EI) vs the Standard Therapy (ST) groups (MD = 1.34; 95% CI, − 5.55 – 8.24).

**Fig. 3:**
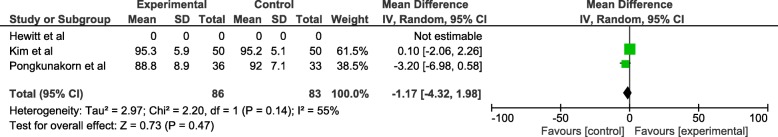
Forest plot diagram of the Knee Society Score, Exercise Intervention (EI) vs Standard Therapy (ST)

**Fig. 4:**
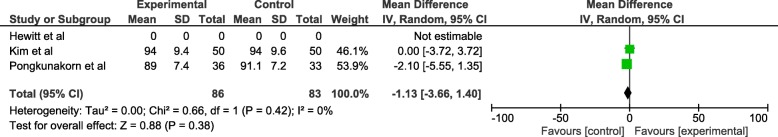
Forest plot diagram of The Knee Society Function Score, Exercise Intervention (EI) vs Standard Therapy (ST)

The Knee Society Score (KSS) and Knee Society Function Score (KSFS) outcomes were reported to be assessed in 3 out of the 4 studies reviewed [27–29], however, only 2 of the 3 studies included the results [27, 29], and as such only 2 data sets are represented in Figs. 3 and 4. The meta-analysis of those 2 studies which included 199 participants are presented. There were no significant differences between the Exercise Intervention (EI) vs the Standard Therapy (ST) groups in either of the Knee Society Score or Knee Society Functions scores, KSS (MD = − 1.17; 95% CI, − 4.32 – 1.98) and KSFS (MD = − 1.13; 95% CI, − 3.66 – 1.40) respectively.

### Methodological quality

Results of the methodological quality assessment, modified from the Downs and Black’s checklist, are presented in Table 6. The methodological quality of the included studies in this review was variable, ranging from 18 to 22 points out of a possible 27, meaning overall the studies were of moderate quality.

**Table 6:** Methodological quality of studies reviewed (Modified Downs & Black)

Author	Study Design	Reporting										External Validity			
		1	2	3	4	5	6	7	8	9	10	11	12	13	
Dujin, P., Jeonghee, K., & Hyunok, L.	Randomised Controlled Trial	1	1	1	1	1	1	1	0	1	0	0	0	1	
Hewitt, B., & Shakespeare, D.	Prospective non-randomised controlled trial (Quasi-Experimental)	0	0	0	1	1	1	1	1	1	1	1	1	1	
Kim, T., Park, K., Yoon, S., Kim, S., Chang, C., & Seong, S.	Randomised Controlled Crossover Trial	1	1	1	1	1	1	1	1	1	1	0	0	1	
Pongkunakorn, A., & Sawatphap, D	Prospective non-randomised controlled trial (Quasi-Experimental)	1	1	1	1	1	1	1	1	1	1	0	0	1	
Author	Internal Validity Bias							Internal Validity Confounding						Power	Total Score
	14	15	16	17	18	19	20	21	22	23	24	25	26	27	
Dujin, P., Jeonghee, K., & Hyunok, L.	0	0	1	1	1	1	1	1	1	1	0	1	1	1	20
Hewitt, B., & Shakespeare, D.	0	0	1	1	1	1	0	1	1	0	0	1	1	0	18
Kim, T., Park, K., Yoon, S., Kim, S., Chang, C., & Seong, S.	0	1	1	1	1	1	1	1	1	0	0	1	1	1	22
Pongkunakorn, A., & Sawatphap, D	0	0	1	1	1	1	1	1	0	0	0	1	1	1	20

Only Kim et al. demonstrated strong methodological quality, scoring 22/27, however, external validity was high for gender in that the subjects were all female and therefore not a true representation of the entire recruitment population for TKR surgery. All studies apart from Hewitt and Shakespeare reported clear objectives, outcomes and included a power calculation. Of the four included trials, all either made no attempt or it was unable to determine if the subjects were blinded to the intervention group they had been allocated to, or whether the randomized intervention assignment was concealed from patients and health care staff until recruitment was completed. There were no adverse events reported across all studies and losses to follow up were minimal and reported on.

## Discussion

### Main findings

The main goal of the present systematic literature review and meta-analysis was to determine the effects of early exercise therapy on patient reported and functional outcomes in a post-operative primary total knee replacement population. Although individual significant differences between therapy groups are noted, when combined for meta-analysis no significant differences between physiotherapy groups were found across Maximum Knee Flexion or Knee Society Scores at 6 weeks. The systematic review included four studies of varying design examining four different supervised exercise therapy programs following TKR surgery in the early post-operative setting. Participant inclusion and exclusion criteria were similar across all studies and sample sizes were appropriately powered to determine significance of the chosen outcomes measured. True randomisation of group allocation did not occur in two of the trials, both having a prospective controlled trial design. Methodological quality assessment of the studies reviewed were of moderate quality, hence the systematic literature review findings should be interpreted accordingly.

Although the Modified Quadriceps Setting exercise patients showed a greater hamstring and gluteal muscle strength the study did not assess knee ROM or include a PROM tool and therefore could not be included in the meta-analysis [26]. A Passive Range of Motion Exercise (PROME) performed by a physiotherapist does not offer additional clinical benefits to standard active exercise therapy to patients after TKR [27], however, positioning a patient in a flexion splint for the first 48 h post-operatively showed greater knee flexion ROM at 6 weeks than an extension splint combined with active flexion exercises [28]. When compared to CPM plus standard physiotherapy, an active-assist Drop and Dangle knee flexion exercise results in increased knee flexion ROM in the first 2 post-operative days following TKR surgery, however, these differences were no longer significant at 6 weeks [29].

### Strengths and limitations of the review

The present review has strength in its thorough search strategies based on the PRISMA guidelines (Additional file 2), its systematic nature and the use of high-quality analysis tool that have high internal consistency and criterion validity, good test-retest reliability and high inter-rater reliability.

Limitations to the review were the lack of randomized controlled trials available on the topic, and as such, quasi-experimental trials were included in the search criteria to broaden the results. The study selection and data extraction were made by the primary investigator which could lead to selection bias of the included studies. The meta-analysis was limited to including those outcomes which were present across 2 or more studies and, consequently, only outcomes that were assessed from 6 weeks and beyond post-operatively could be investigated, thereby not including any treatment effects in the early inpatient phase. The considerable clinical heterogeneity of the exercise interventions investigated in each of the included studies also makes it difficult to guide best evidence-base practice for exercise therapy early after TKR.

### Clinical and research implications

The small number of heterogeneous studies identified precludes the formulation of clinical guidelines as to the optimum type, frequency or duration of early exercise therapy after TKR. Given the cost of providing these inpatient services, it is surprising that such a large deficit exists in the literature. In contradistinction, a recent Cochrane review of CPM, identified 24 randomised controlled trials of CPM with standard postoperative care compared to similar postoperative care [30]. There is a need for further studies of high-quality design into supervised exercise therapy programs to provide greater functional outcomes and patient reported satisfaction following TKR surgery, particularly in the early post-operative period.

## Conclusion

Accelerated discharge pathways following TKR are becoming increasingly popular and consequently hospital LOS rates are declining. This review demonstrates that there are few studies available on early supervised exercise therapy following TKR surgery in the immediate post-operative setting, with a heterogeneous group of exercises examined. The lack of large randomised trials with adequate methodology on physiotherapy for TKR patients in the early post-operative period highlights the need for further research of higher quality design.

## Additional files

Additional file 1: Modified Downs and Black Checklist. For the assessment of the methodological quality of both randomized and non-randomized studies. (DOCX 21 kb)

Additional file 2: PRISMA Checklist. The 27 checklist items pertain to the content of a systematic review and meta-analysis, which include the title, abstract, methods, results, discussion and funding. (DOC 63 kb)

## Abbreviations

CPM: Continuous passive movement
EI: Exercise intervention
KSFS: Knee society function score
KSS: Knee society score
LOS: Length of stay
OA: Osteoarthritis
PRISMA: Preferred reporting items for systematic reviews and meta-analyses
PROM: Patient reported outcome measures
PROME: Passive range of motion exercise
ROM: Range of motion
SD: Standard deviation
ST: Standard therapy
TKR: Total knee replacement
